# Meckel’s Diverticulum Charading as Crohn’s Disease: A Single-Institution Case Series

**DOI:** 10.7759/cureus.38191

**Published:** 2023-04-27

**Authors:** Nina L Eng, Audrey Kulaylat, Nimalan A Jeganathan, Jeffery S Scow, Michael Deutsch

**Affiliations:** 1 General Surgery, Penn State Health Milton S. Hershey Medical Center, Hershey, USA; 2 Colorectal Surgery, Penn State Health Milton S. Hershey Medical Center, Hershey, USA

**Keywords:** inflammatory bowel disease (ibd), crohn’s disease (cd), meckel’s diverticulum, colorectal diseases, meckel’s diverticulitis

## Abstract

Meckel’s diverticulum is the most common gastrointestinal congenital anomaly and may present with lower gastrointestinal bleeding, abdominal pain, and nausea. Imaging and endoscopic findings can be similar to those of Crohn’s disease, including transmural inflammation, stricturing, and superficial ulceration frequently in the distal ileum. Here, we present a case series of three patients who were initially diagnosed with Crohn’s disease and ultimately found to have Meckel’s diverticulum alone on final pathology. This single-institution case series, the largest in the literature, highlights the importance of maintaining a high index of suspicion for Meckel’s diverticulum, especially in the absence of microscopic evidence of inflammatory bowel disease.

## Introduction

Meckel’s diverticulum (MD), first described by Wilhelm Fabricius Hildanus in 1598, is an intestinal outpouching that forms due to the incomplete obliteration of the omphalomesenteric duct, most often occurring within 100 cm of the ileocecal valve [1,2-7]. Considered the most common gastrointestinal congenital anomaly, it is estimated to occur in 0.4-2% of the general population and is often incidentally diagnosed [1-3]. The most common presentations in adults over the age of 40 are obstruction and inflammation, while lower gastrointestinal bleeding is more commonly seen in adults under the age of 40 and in children [4,8].

Diagnosis of MD is achieved with a thorough patient history and physical examination as well as careful review of imaging, typically contrast-enhanced computerized tomography (CT) in adults and 99mTc-Na-pertechnetate scintigraphy, or a “Meckel’s scan,” in children. Although the sensitivity of CT is high in most symptomatic cases, small MD can be difficult to detect when the surrounding bowel is inflamed and can mimic other inflammatory conditions, including Crohn’s disease [3-9]. The standard treatment of symptomatic MD is resection of the diverticulum and any associated abnormal tissue. Misdiagnosis may result in a delay in surgery and the administration of non-indicated medications and interventions.

In this case series, we discuss three adult patients who underwent medical therapy and surgery for presumed terminal ileal Crohn’s disease and were found to have MD alone on final pathology.

## Case presentation

Patient one

**Figure 1:**
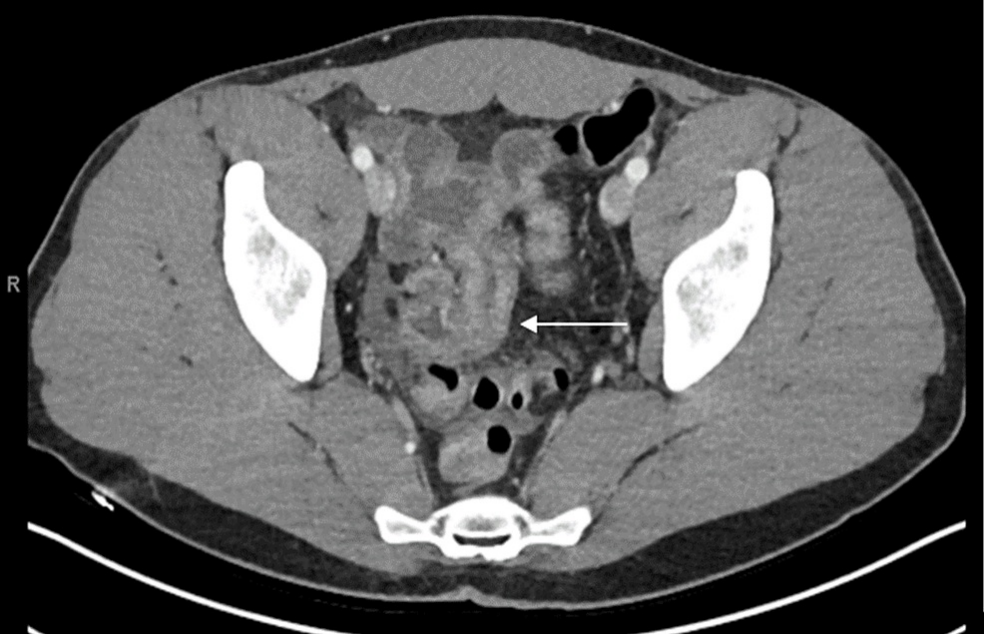
Preoperative computerized tomography imaging demonstrating severe terminal ileitis (white arrow) initially concerning for Crohn’s disease.

A 32-year-old otherwise healthy male presented to his primary care provider with dull, cramping abdominal pain. The pain sharply worsened the next day, and he presented to the emergency department with additional new-onset nausea and diarrhea. Labs were notable for a mild leukocytosis, a mildly elevated C-reactive protein (CRP), normal erythrocyte sedimentation rate (ESR), and elevated fecal calprotectin of 374. CT imaging of the abdomen and pelvis with intravenous (IV) contrast was notable for small bowel dilation with two segments of inflammation and severe terminal ileitis concerning for Crohn’s disease (Figure 1). Gastroenterology (GI) was consulted, and he underwent a colonoscopy, which was notable for nodular ileal mucosa. The final pathology of the biopsies demonstrated normal mucosa without pathologic alteration. He was initiated on a prednisone taper for presumed Crohn’s disease and referred to the inflammatory bowel disease (IBD) clinic, where he was initiated on adalimumab therapy. Four months later, he presented again to the emergency department with severe obstructive symptoms, and CT imaging noted narrowing in the terminal ileum and dilated proximal small bowel concerning for a partial small bowel obstruction. Magnetic resonance enterography (MRE) demonstrated increased terminal ileum wall thickening and mucosal enhancement with signs of upstream small bowel obstruction. He was treated non-operatively with bowel rest, high-dose steroids, and antibiotics and discharged with short interval follow-up with gastroenterology and colorectal surgery.

The patient underwent elective diagnostic laparoscopy, and intraoperative findings included stricturing of the terminal ileum and an adjacent MD. A robotic-assisted ileocolectomy was performed, and the final pathology of the specimen was notable for an MD and otherwise normal mucosa. He recovered unremarkably with full resolution of his symptoms, and adalimumab was stopped. Three months later, he underwent a colonoscopy which was notable for normal terminal ileum and colon without any signs of Crohn’s disease.

Patient two

A 56-year-old male with a history of gastric arteriovenous malformations and chronic anemia requiring prior endoscopic interventions was evaluated in the GI clinic for ongoing anemia as well as intermittent episodes of abdominal pain with nausea. He underwent video capsule endoscopy which was notable for distal jejunal stenosis and distal ileal ulcerations. He also underwent CT enterography, which demonstrated the video capsule within the proximal dilated small bowel and mid-to-distal ileal luminal narrowing. These findings were thought to be concerning for Crohn’s disease, and he was initiated on a prednisone taper. Labs at the time were notable for mild anemia, normal white blood cell count, normal CRP and ESR, and an elevated fecal calprotectin level of 362. Upper and lower endoscopy as well as retrograde double-balloon enteroscopy (DBE) were performed and were notable for benign-appearing strictures in the distal jejunum and shallow ulcerations with surrounding inflammation at the distal ileum. The final pathology of all biopsies noted normal small intestinal mucosa without dysplasia. He was presented at a multidisciplinary IBD conference, diagnosed with presumed fibrostenotic Crohn’s disease, and referred for colorectal surgery evaluation for resection of the strictured bowel. After a discussion with the patient, the decision was made to proceed with surgical intervention.

A diagnostic laparoscopy was performed, and upon evaluation of the small bowel laparoscopically and extracorporeally, there were no identified strictured segments of the bowel or external findings suggestive of Crohn’s disease. The only abnormality noted was an MD in the distal ileum with associated adjacent inflammation and mild proximal small bowel dilation, and this section of the ileum was resected. The final pathology of the specimen demonstrated MD with focal gastric heterotopia and patchy mild ileitis. He recovered well postoperatively, and his symptoms resolved completely. A CT scan performed one year postoperatively demonstrated resolution of his previously seen ileal luminal narrowing.

Patient three

**Figure 2:**
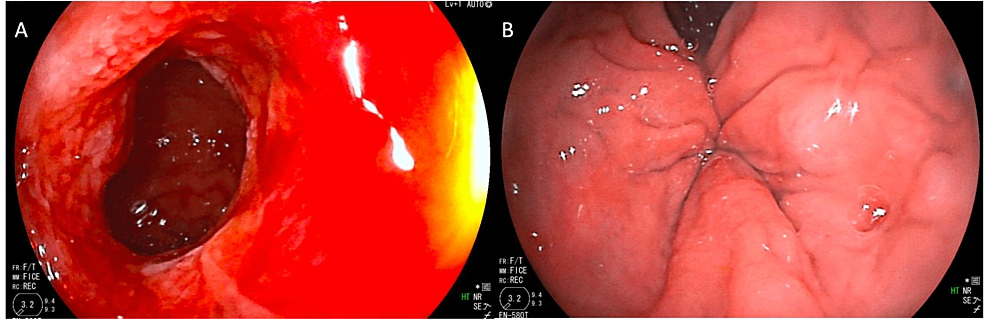
Representative images captured during double-balloon enteroscopy demonstrating adjacent areas of (A) inflammation and (B) stricturing of the distal small bowel.

A 63-year-old male with a history of hypertension, atrial fibrillation, and obstructive sleep apnea, was referred to GI after experiencing five episodes of severe obstructive symptoms over a four-year time period requiring repeat hospitalizations for nasogastric decompression. During this time, he underwent numerous CT and magnetic resonance imaging (MRI) scans notable for transition points in the distal ileum. These findings were initially suspicious for Crohn’s disease, and GI performed retrograde DBE, which demonstrated two segments of distal ileum stricturing with active ulcerations (Figure 2). Biopsies demonstrated mild-to-moderate, active ileitis with reactive lymphoid aggregates, and no dysplasia. His labs at the time of initial presentation were unremarkable including a normal CRP level and absent leukocytosis. Fecal calprotectin was not evaluated. The patient was diagnosed with Crohn’s disease and initiated on mesalamine therapy, as the patient refused first-line adalimumab. He was referred for colorectal surgery evaluation, and the initial plan was to avoid surgical intervention given a relative improvement in his symptoms with mesalamine.

Eight months later, the patient underwent scheduled, repeat retrograde DBE, which re-demonstrated the known ulcerated and stricturing areas of the distal ileum. Dilation of the strictures was performed, and biopsies demonstrated chronic ileitis. Post-procedurally, the patient developed severe lower abdominal pain and nausea and was referred to the emergency department. CT imaging of the abdomen and pelvis with IV contrast did not demonstrate obstruction or signs of perforation. He was discharged after symptoms improved with bowel rest and nasogastric decompression and was ultimately transitioned to adalimumab therapy two months later for recurrent obstructive symptoms.

He was evaluated again by colorectal surgery nine months later and was scheduled for elective surgery. He underwent diagnostic laparoscopy and laparoscopic ileocolectomy, which was notable for mild terminal ileum inflammation and an adjacent MD. The final pathology of the specimen confirmed an MD, lymphoid aggregates within the terminal ileum, and no microscopic signs of Crohn’s disease. Postoperatively, the patient’s Crohn’s disease therapy was stopped, and all his symptoms have since resolved.

## Discussion

Diagnosis of MD is frequently challenging and was described by Dr. Charles W. Mayo as being “frequently suspected, often looked for, and seldom found” [10]. In this case study, we present three cases highlighting adults with symptomatic MD who were diagnosed with suspected Crohn’s disease and treated initially as such. CT with IV contrast is most commonly utilized, though as evidenced by our patients, can miss small MD and/or those with surrounding inflamed or strictured bowel [2-4]. MD may contain heterotopic mucosa, including gastric and pancreatic tissue, though this is identified in less than half of symptomatic adults, reducing the sensitivity of 99mTc-Na-pertechnetate scintigraphy [2]. Angiography can be helpful in cases of gastrointestinal bleeding, though its utility is limited to patients with active bleeding of at least 0.5 mL/minute, which is a rare presentation of MD in the adult population [11]. Other diagnostic modalities including MRI, CT or MR enteroclysis, upper/lower endoscopy, and barium enemas offer varying success rates in the diagnosis of MD and may further suggest a diagnosis of Crohn’s disease in patients with terminal ileum inflammation or structuring [11]. Ultimately, surgical exploration is the most definitive diagnostic and therapeutic modality for symptomatic MD.

In addition to an overlap of symptoms between Crohn’s disease and MD, some data suggest MD is more commonly found in patients with Crohn’s disease compared to the general population, though the majority of data consists of isolated case reports [9,12-15]. The largest retrospective review to note an association was published by Andreyev et al. (1994), who reviewed 294 consecutive patients with Crohn’s disease who underwent right hemicolectomy and found the prevalence of MD to be 5.8% [9]. In contrast, Freeman (2001) more recently reviewed 877 patients with Crohn’s disease and noted an overall MD prevalence of 1%. Of the patients who required a bowel resection for Crohn’s disease, 2% were noted to have an MD, similar to the prevalence described among the general population [16]. A common underlying factor that would predispose patients to both has not been reliably identified, though some hypotheses include acid secretion from gastric mucosa within MD causing adjacent bowel inflammation, increased bowel permeability due to trapped microbes within the diverticulum, and disruption in gut motility and lymphatic drainage [15].

## Conclusions

The gold standard of care for MD is surgical resection, ranging from simple diverticulectomy to segmental bowel resection in cases of active inflammation or obstruction. This single-institution case series, the largest in the literature, demonstrates three examples of delay in treatment as well as the performance of numerous imaging studies and procedures and administration of inappropriate medications ranging from steroids to immunotherapy. The authors hope these cases highlight the importance of maintaining a high index of suspicion for MD, particularly in the absence of microscopic evidence of IBD.
